# Cotton Cationization and Dyeing in One Bath: A Short-Process, Salt-Free Approach for Reactive Dyes

**DOI:** 10.3390/polym18151867

**Published:** 2026-07-30

**Authors:** Zijian Dai, Jiao Li, Zixian Zhang, Huiqiang Wang, Jianyong Yu, Hongjuan Zhang, Yang Si

**Affiliations:** 1 Innovation Center for Textile Science and Technology, Donghua University, Shanghai 200051, China; zjdai@dhu.edu.cn (Z.D.); kjsd165@163.com (J.L.); yujy@dhu.edu.cn (J.Y.); 2 School of Textile and Fashion, Shanghai University of Engineering Science, Shanghai 201620, China; 13462991136@163.com (Z.Z.); m395123101@sues.edu.cn (H.W.); 3 State Key Laboratory for Modification of Chemical Fibers and Polymer Materials, Donghua University, Shanghai 201620, China

**Keywords:** cotton fabrics, cationic modification, reactive dyes, salt-free dyeing, cleaner production

## Abstract

To promote the sustainable development of the textile industry and address the critical challenges of high water consumption and salt pollution in cotton dyeing, this study developed a short-process cationic dyeing method using 2,3-epoxypropyltrimethylammonium chloride (GTA). Unlike traditional cationic dyeing, this approach eliminates the intermediate drying step by adding reactive dyes directly into the same bath after modification, achieving salt-free dyeing and significant water savings. The effects of key parameters, including GTA concentration, NaOH concentration, modification temperature and time, dyeing bath ratio, holding time, and dyeing temperature, were systematically investigated. The optimal dyeing conditions for C.I. Reactive Red 195, Reactive Black 5, and Reactive Blue 19 were determined as follows: GTA concentration of 40 g/L, NaOH concentration of 1 g/L, modification at 90 °C for 50 min, dyeing at 60 °C with a bath ratio of 1:15, and holding times of 30, 20, and 40 min, respectively. After modification, thermal stability and crystallinity decreased slightly (from 60.51% to 58.18%), while hydrophilicity showed no significant change. This short-process cationic dyeing method saves time, energy, and water while maintaining dyeing performance comparable to conventional methods. It provides a practical and sustainable new approach for salt-free cotton dyeing.

## 1. Introduction

Given its comfort, safety, renewability, and wide applicability in apparel and medical textiles, cotton remains an irreplaceable and sustainable natural fiber that serves as a key alternative to synthetic materials [1,2,3]. However, despite the significant advantages of cotton fabrics, their traditional dyeing process still suffers from prominent environmental and technical bottlenecks. The cotton fiber surface, rich in hydroxyl groups (–OH), carries a negative charge and creates strong electrostatic repulsion toward anionic dyes (e.g., reactive dyes). To improve dye uptake, conventional processes rely on large amounts of electrolytes, such as sodium chloride or sodium sulfate, to shield the fiber surface charge and facilitate dye adsorption. This approach typically consumes salt at a concentration of 50–100 g/L [4,5,6], leading to the accumulation of salt and unfixed dyes in wastewater, causing water eutrophication, soil salinization, and biological toxicity [7,8,9,10,11,12]. Furthermore, the conventional process requires multiple washing steps to remove unfixed dyes, significantly increasing water and energy consumption. It also suffers from low dye utilization (sometimes below 70%) [13,14,15,16,17,18,19] and insufficient color fastness. These drawbacks severely restrict the green transformation of the cotton textile industry.

To address these pressing issues, the cationic modification of cotton fibers has emerged as one of the most effective strategies [20,21,22,23,24,25,26,27]. This approach involves applying cationic modifiers to the fiber surface via physical adsorption or chemical bonding to increase the positive charge density, thereby counteracting the negative charge of the cotton. Common cationic modifiers typically contain reactive groups such as amino, quaternary ammonium, guanidine, imidazole, or pyridine moieties [28,29,30,31,32,33], enabling them to bind to the fibers via covalent or ionic bonds. However, not all cationic modifiers perform equally. For example, 3-chloro-2-hydroxypropyltrimethylammonium chloride (CHPTAC) can readily diffuse into the interior of fibers but exhibits low fiber affinity, thus requiring a high dosage (usually 50–200 g/L) to achieve salt-free dyeing [34,35,36]. Early studies on small-molecule modifiers such as 3-chloro-2-hydroxypropyl trimethyl ammonium chloride [37,38,39] were limited by their low affinity and impractically high dosage requirements. Consequently, subsequent research has focused primarily on high-molecular-weight cationic polymers (e.g., quaternary ammonium salts and tertiary amine compounds), which can achieve effective modification at a much lower dosage. Most studies indicate that this approach enables salt-free dyeing while matching the exhaustion effect of traditional salt-based processes [40,41,42,43]. For example, Pan et al. [44,45] synthesized a novel chitosan derivative (NMA-HDCC) using chitosan (CTS) with antibacterial activity, glycidyl dimethyl quaternary ammonium salt, and *N*-methylacrylamide (NMA) as raw materials. Treating cotton fibers with this water-soluble derivative achieves salt-free dyeing of cotton fabrics and imparts certain antibacterial properties to them. Dong et al. [46] grafted the pH-responsive cationic polyelectrolyte 2-(*N*,*N*-dimethylamino) ethyl methacrylate (PDMAEMA) onto cotton fabrics. Under salt-free conditions, the dye uptake rate reached 98%, significantly exceeding conventional salt-based dyeing levels. However, this compound is costly to prepare and requires specialized equipment for pretreating cotton fabrics.

To overcome pretreatment modification, long processing time, and uneven dyeing in conventional cationic dyeing of cotton fabrics, this study employed 2,3-epoxypropyltrimethylammonium chloride (commonly known as GTA) at a lower dosage to modify cotton fabrics. After modification, reactive dyes were added directly to the same bath for dyeing. The process was optimized by reducing the NaOH concentration during modification, thereby minimizing dye hydrolysis. In addition, the typically required washing and drying steps after modification were omitted, which shortened the overall dyeing process and reduced water consumption. This short-process cationic dyeing not only achieves salt-free dyeing of cotton fabrics but also saves time and water, offering a promising new approach for future salt-free dyeing of cotton fabrics.

## 2. Materials and Methods

### 2.1. Materials

This study used 100% cotton woven fabric (146 g/m^2^, yarn density: 134 × 257, yarn count: 35 s × 35 s). Sodium hydroxide (NaOH, A.R.) was purchased from Sinopharm Chemical Reagent Co., Ltd.; 2,3-epoxypropyltrimethylammonium chloride (GTA) from Shanghai Macklin Biochemical Technology Co., Ltd.; and C.I. Reactive Red 195, Reactive Black 5, and Reactive Blue 19 from Shanghai Macklin Biochemical Technology Co., Ltd. Their chemical structures are shown in Figure S1.

### 2.2. Dyeing Processes and Instruments

#### 2.2.1. Traditional Salt-Containing Dyeing Process (TSDP)

For Reactive Red 195, the dye concentration was 2% (o.w.f.), and the bath ratio was 1:15. Initially, 1.8 g of sodium sulfate (Na_2_SO_4_) was added to the dye bath, followed by adsorption at 30 °C for 10 min. The temperature was then raised to 90 °C at a rate of 2 °C/min. After 10 min, 0.6 g of sodium carbonate (Na_2_CO_3_) was added, and the temperature was maintained for 60 min. After dyeing, the cotton fabric was rinsed with tap water, washed with 2 g/L soap solution at 95 °C for 15 min, and finally rinsed with tap water to remove unreacted and hydrolyzed dyes. The dyed samples were dried in an oven at 60 °C.

#### 2.2.2. Traditional Salt-Free Dyeing Process (TFDP)

As shown in Figure S2, the cotton fabric was immersed in a cationic modification solution containing 30 g/L GTA and 3 g/L NaOH. The bath ratio was 1:20. The modification was carried out at 90 °C for 20 min; then, the fabric was washed and dried. The modified cotton fabric was immersed in the dye bath (with the same dye concentration as the traditional salt-containing dyeing process) at room temperature for 30 min. The temperature was then raised to 60 °C at a rate of 2 °C/min and maintained for 60 min. After dyeing, the cotton fabric was rinsed with tap water, washed with 2 g/L soap solution at 95 °C for 15 min, and finally rinsed with tap water to remove unreacted and hydrolyzed dyes. The dyed samples were dried in an oven at 60 °C.

#### 2.2.3. Short-Process Salt-Free Dyeing Process (SSFP)

Based on the traditional cationic dyeing process, dye solution was added directly to the above-mentioned modified solution to realize the short-process dyeing (Figure S2). The effects of several key parameters on dyeing performance were systematically investigated, including GTA and NaOH concentration, modification time, modification temperature, dyeing bath ratio, holding time, and dyeing temperature. Process formulation: the dye dosage was the same as that in the TSDP; the GTA concentrations were 5, 10, 20, 30, 40, 50, 60 g/L; NaOH concentrations were 0.5, 1, 2, 3, 4, 5 g/L; modification times were 10, 20, 30, 40, 50, 60 min; modification temperatures were 70, 80, 90, 95, 100 °C; dyeing bath ratios were 1:5, 1:10, 1:15, 1:20, 1:30, 1:40; holding times were 10, 20, 30, 40, 50, 60 min; and dyeing temperatures were 40, 50, 60, 70, 80, 90 °C.

#### 2.2.4. Instruments

A Dye-24 Adjustable Dyeing Machine (Shanghai Qianli Automation Equipment Co., Ltd., Shanghai, China); a Datacolor 800 (Datacolor Trading Shanghai Co., Ltd., Shanghai, China); an L-24 Oscillating Water Bath (Xiamen Ruibi Precision Machinery Co., Ltd., Xiamen, China); a J-20 Dehydrator (Shaoxing Hongjing Textile Machinery Equipment Co., Ltd., Shaoxing, China); and a DHG-9070A Electric Thermostatic Blast Drying Oven (Shanghai Yiheng Scientific Instrument Co., Ltd., Shanghai, China) were used.

**Figure 1:**
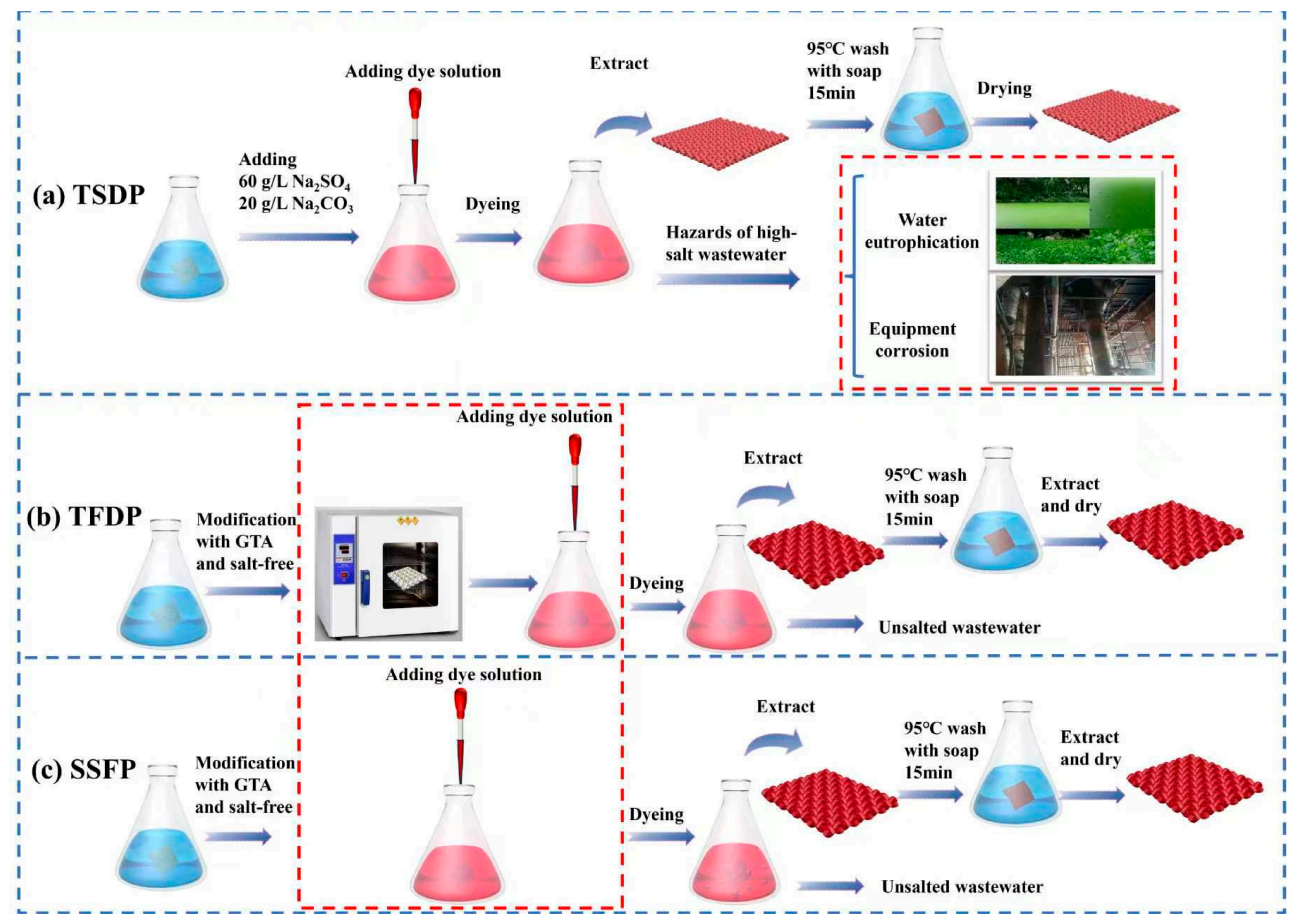
Schematic diagrams of (**a**) traditional salt-containing dyeing process (TSDP), (**b**) traditional salt-free dyeing process (TFDP), and (**c**) short-process salt-free dyeing process (SSFP). The red frame shows the intermediate drying step by adding reactive dyes directly into the same bath after modification for SSFP compared with TFDP.

An overview of the three dyeing routes and a brief comparison are presented in Figure 1.

### 2.3. Process Investigation

#### 2.3.1. Investigation of Optimal Modification Process

The effect of different GTA concentrations on the modification performance of cotton fabrics was investigated under a NaOH concentration of 3 g/L. The effect of different NaOH concentrations on the modification performance of cotton fabrics was investigated under a GTA concentration of 40 g/L. The effect of different modification times on the modification performance of cotton fabrics was investigated under the GTA concentration of 40 g/L and NaOH concentration of 1 g/L. The effect of different modification temperatures on the modification performance of cotton fabrics was investigated under the conditions of 40 g/L GTA, 1 g/L NaOH, and a modification time of 50 min.

#### 2.3.2. Investigation of Optimal Dyeing Process

The effect of different dyeing bath ratios on the dyeing properties of cotton fabrics was investigated according to the process in Section 2.3.1. The effect of different holding times on the dyeing properties of cotton fabrics was investigated in a dyeing bath ratio of 1:15. The effect of different dyeing temperatures on the dyeing properties of cotton fabrics was investigated under the conditions of a dyeing bath ratio of 1:15 and a holding time of 30 min.

### 2.4. Characterization

To determine whether GTA was successfully grafted onto the cotton fabrics after modification, FT-IR analysis was conducted using the KBr pellet method. Spectra of the fabrics before and after treatment were recorded in the wavenumber range of 400–4000 cm^−1^, at a resolution of 4 cm^−1^ with 32 scans. XRD was used to analyze the cotton fabrics before and after modification, with a scanning range of 10–60°, a scanning speed of 4°/min, a step size of 0.02°, and an X-ray wavelength of 1.5406 nm. The diffraction patterns were recorded in the 2θ range. A Pyris Thermogravimetric Analyzer (TGA) (Pyris, PerkinElmer, MA, USA) was used to analyze the effect of GTA on the thermal properties of cotton fabrics. Under a nitrogen flow of 20 mL/min, the samples were heated from 30 °C to 600 °C at a heating rate of 10 °C/min. The Zeta potential of GTA-modified cotton and raw cotton is measured to verify the success of the modification through changes in surface charge. Briefly, 0.5 g of cotton fabric samples were accurately weighed before and after dye fixation and then cut into 1 mm short fibers and immersed in a KCl solution of 0.001 mol/L. After ultrasonic dispersion for 10 min to prepare a uniform suspension, the supernatant was collected after centrifugation and measured using a Zeta potential analyzer (BeNano 180 Zeta Max, Dandong, China). Fabric water absorption rate, water droplet wetting time, and capillary rise height were tested according to GB/T 21655.1-2023 “Textiles—Evaluation of absorption and quick-drying—Part 1: Method for combination tests [47].” The K/S value at the maximum absorption wavelength of the dyed fabrics was determined by using a Datacolor 800 color measuring and matching instrument and measured at five different locations for each dyed fabric. The color parameters were tested in a Datacolor 800 spectrophotometer with the CIE Illuminant D65 using the 10° standard observer in the visible spectrum region of 400 to 800 nm. The levelness properties of dyed fabrics were evaluated by the ΔE value.

### 2.5. Determination of Dye Uptake Rate and Fixation Rate

According to the dyeing process shown in Figure 1, cotton fabrics were dyed with C.I. Reactive Blue 19, Reactive Black 5, and Reactive Red 195, respectively. The residual dyeing solution and residual washing solution were collected. The residual dyeing solution was diluted, and its absorbance was measured and recorded. The dye uptake rate (E%) and fixation rate (F%) are basic parameters for evaluating the dyeing effect, which were calculated according to Equations (1) and (2):

(1) $$E\;(\%)=\frac{{n_{0}A}_{0}-{n_{1}A}_{1}}{{n_{0}A}_{0}}\times 100\%$$

(2) $$F\;\left(\%\right)=\frac{{n_{0}A}_{0}-{n_{1}A}_{1}-n_{2}A_{2}}{n_{0}A_{0}}\times 100\%$$

where A_0_, A_1_, and A_2_ are the absorbances of the original dye solution, the residual dye solution after dyeing, and the soap solution, respectively, and n_0_, n_1_, and n_2_ are the dilution factors. A UV–Vis spectrophotometer (UV-2600 UV–Vis Spectrophotometer, Shimadzu, Kyoto, Japan) was used to measure the absorbance at the maximum absorption wavelength (λmax) of the dye, and the calculation was performed according to the Lambert–Beer law.

### 2.6. Analysis of the Binding Mode Between Reactive Dyes and Modified Cotton Fabrics

#### 2.6.1. Preparation of Hydrolyzed Reactive Dye Solution

To confirm whether covalent bonds were formed between GTA-modified cotton and dyes, a 10 g/L dye stock solution was first prepared, adjusted to pH 11, and heated to 90 °C for 4 h of incubation. The hydrolyzed dye was then used to dye the modified cotton fabrics. Preparation of hydrolyzed samples for traditional water bath dyeing: The dye dosage was 2% (o.w.f.); the bath ratio was 1:20; and the dye solution concentration was 1 g/L. Weigh 0.1 g of dye and dissolve it in 100 mL of buffer solution with pH 11 at a specific temperature. Immediately start timing, sample 5 mL at regular intervals, dilute to 25 mL with buffer solution of pH 4, and filter for later use.

#### 2.6.2. Preparation of Buffer Solutions

Alkaline buffer solution (pH = 11): 8.40 g of sodium bicarbonate and 152.32 g of sodium carbonate were weighed and dissolved in 500 mL of deionized water and finally diluted to 1000 mL. Acidic buffer solution (pH = 4): 32.79 g of sodium acetate and 92 mL of acetic acid were weighed and dissolved in 500 mL of deionized water and finally diluted to 1000 mL.

#### 2.6.3. High-Performance Liquid Chromatography (HPLC) Analysis

An XBridge^®^ C18 reverse-phase chromatographic column (Wexford, Ireland, 5 μm, 4.6 mm × 250 mm) was used with gradient elution, a flow rate of 1 mL/min, an injection volume of 10 μL, and a column temperature of 30 °C. Mobile phase A was a mixed aqueous solution of 0.002 mol tetrabutylammonium bromide and 0.05 mol/L ammonium acetate; mobile phase B was HPLC-grade acetonitrile solution. The gradient elution conditions for reactive dyes with different structures are shown in the following Table 1, Table 2 and Table 3.

C.I. Reactive Red 195 was detected by a diode-array detector (DAD) at a detection wavelength of 542 nm.

C.I. Reactive Black 5 was detected by a DAD at a detection wavelength of 598 nm.

C.I. Reactive Blue 19 was detected by a DAD at a detection wavelength of 592 nm.

### 2.7. Color Fastness of Dyed Cotton Fabrics

The rubbing fastness was tested according to ISO 105-X12:2016 “Textiles—Tests for color fastness—Part X12: Color fastness to rubbing” on the Y571 Rubbing Fastness Tester (Laizhou Yuanmao Instrument Co., Ltd.) [48]. The washing fastness was tested according to ISO 105-C03:2010 “Textile—Tests for color fastness—Part C03: Color fastness to washing” on the SW20A-IIA Washing Fastness Tester (Ningbo Textile Instrument Factory) [49].

### 2.8. Statistical Analysis

All data presented in this study were derived from a minimum of three independent experiments and are expressed as the mean value ± standard deviation. Statistical analysis was conducted using one-way analysis of variance (ANOVA) through IBM SPSS Statistics 22.0 software. All statistical tests established significance levels at * *p* ≤ 0.05, ** *p* ≤ 0.01 and *** *p* ≤ 0.001 relative to the optimal group in each test.

## 3. Results and Discussion

### 3.1. Characterization and Mechanism Analysis of GTA-Modified Cotton

#### 3.1.1. Characterization of Modified Cotton

**Figure 2:**
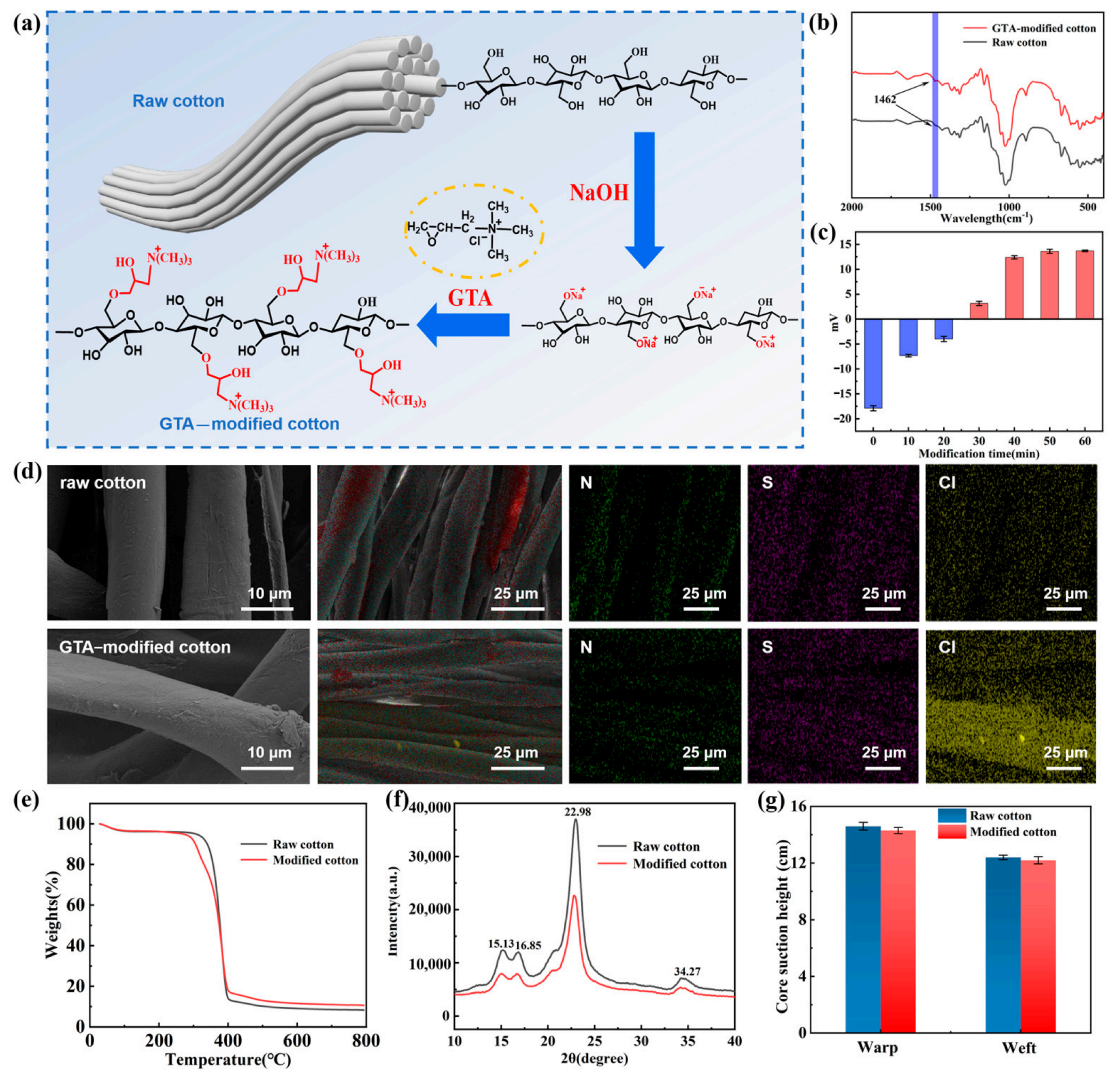
(**a**) Mechanism analysis diagram of GTA-modified cotton. (**b**) FT-IR spectra of unmodified cotton and GTA-modified cotton. (**c**) Zeta potential of unmodified cotton and GTA-modified cotton at different times. (**d**) SEM and EDS images of cotton fabrics and GTA-modified cotton. Physical property analysis of cotton fabrics before and after modification: (**e**) thermogravimetry; (**f**) XRD patterns; (**g**) wick height in different directions.

The mechanism of the GTA-grafted cotton fabric process is illustrated in Figure 2a. In this process, under alkaline conditions, the epoxy ring of GTA opens and reacts with the C6 hydroxyl group of the cotton fabric, simultaneously generating a β-hydroxyl group. In general, the hydrophilicity of the cotton fabric does not change significantly before and after GTA modification. Because the surface of the GTA-grafted cotton fabric contains both cationic groups and hydroxyl groups, reactive dyes can form both ionic and covalent bonds with the fabric during the dyeing process. Figure 2b shows the FT-IR spectra of unmodified cotton and GTA-modified cotton after grafting. In the spectrum of unmodified cotton, the bending vibration peak of O-H appears at 1645 cm^−1^, and the vibration peaks of methylene C-H and methyl C-H are observed at 1428 cm^−1^ and 1312 cm^−1^. In addition, the peak at 1048 cm^−1^ is assigned to the stretching vibration of C-O-C. These characteristics are typical of the infrared spectrum of cotton fibers. Compared with unmodified cotton, a new peak was found at around 1462 cm^−1^, which corresponds to the stretching vibration of quaternary ammonium groups (C-N) for GTA-modified cotton. These results indicate that GTA has been grafted onto the cotton surface. As shown in Figure 2c, the surface potential of unmodified cotton fabric is −17.9 mV. It was clearly found that the fabric potential shows a regular positive shift and that the charge reversal (positive state) is completed after the modification time of 30 min. When the modification time exceeds 50 min, the potential tends to be stable, indicating that the grafting of the modifier on the fiber surface tends to be saturated (Figure 2a). As shown in Figure 2d, the SEM images of raw cotton fibers exhibited a smooth surface with natural longitudinal grooves. After GTA modification, the fiber surface showed slight roughness without structural damage, indicating successful surface modification. EDS elemental mappings further verified the modification and dyeing process. For raw cotton, no characteristic signals of N, S, and Cl were detected, which is consistent with the cellulose composition of natural cotton. After GTA modification, uniform distribution of N and Cl elements was observed on the fibers, directly proving the successful grafting of GTA cationic groups. The nitrogen content (*N*%) increased from 0% (for the untreated cotton) to 1.12 wt% after modification (Table S2) at a GTA concentration of 40 g/L. The grafting efficiency of GTA was calculated to be 13.8% based on the nitrogen content.

#### 3.1.2. Physical Property Analysis of GTA-Modified Cotton

The effects of GTA modification on the thermal stability, crystalline structure, and surface hydrophilicity of cotton fabrics were systematically investigated. As shown in Figure 2e, the thermogravimetric curves of cationic cotton and unmodified cotton are different. The weight loss temperature of cationic cotton is 270 °C, which is lower than that of unmodified cotton (300 °C). This is because the thermal stability of cotton is related to its crystallinity, and cotton with high crystallinity has higher thermal resistance than that with low crystallinity. The lower thermal stability of cationic cotton is due to the chemical reactions and hydrogen bond breakage during the modification process, which reduce its crystallinity. Figure 2f shows the XRD patterns of unmodified cotton and GTA-modified cotton. Both samples exhibit the typical crystalline form of cellulose I, with four distinct diffraction peaks located at 15.13°, 16.85°, 22.98°, and 34.27°, respectively. Therefore, the crystalline form of cotton remains unchanged after grafting, indicating that the grafting reaction occurs in the amorphous region of cotton. Notably, the peak intensities of the modified cotton decrease slightly. The crystallinity calculated using Jade 6.5 software (Materials Data, Inc., Livermore, CA, USA) shows that the crystallinity decreases from 60.51% (unmodified cotton) to 58.18% (GTA-modified cotton), which is attributed to the use of NaOH during the modification process. As shown in Figure 2g, the overall hydrophilicity of cotton fabrics does not change significantly before and after modification. This is because, when GTA reacts with the C6 hydroxyl group of cotton, a new hydroxyl group is generated. Additionally, the introduced quaternary ammonium salt groups are also hydrophilic. Consequently, no significant change in hydrophilicity is observed after modification.

### 3.2. Analysis and Determination of Modification Parameters for Different Reactive Dyes

**Figure 3:**
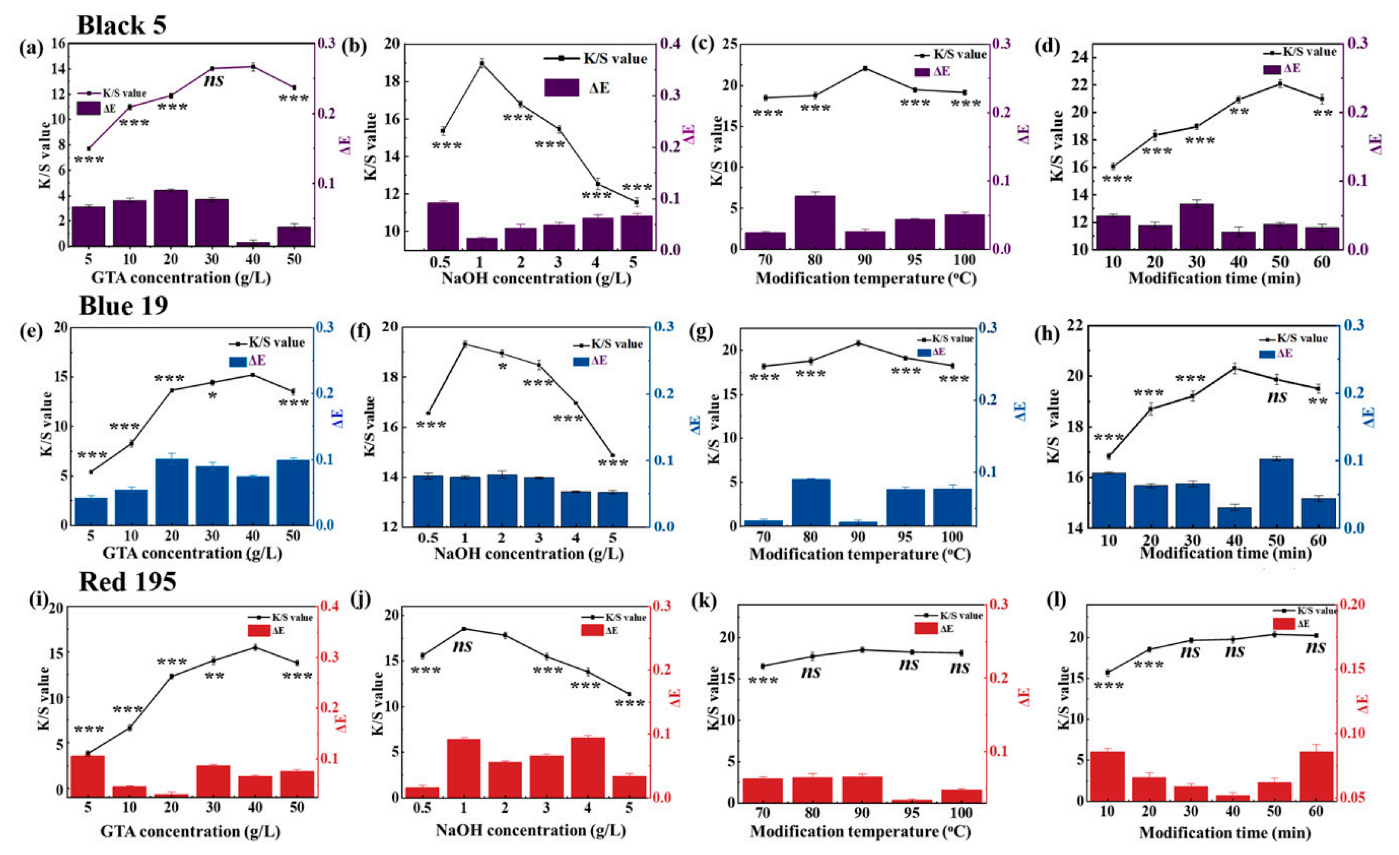
Effects of (**a**,**e**,**i**) GTA concentration, (**b**,**f**,**j**) NaOH concentration, (**c**,**g**,**k**) modification temperature and (**d**,**h**,**l**) modification time on the dyeing performance of C.I. Reactive Black 5, Reactive Blue 19 and Reactive Red 195. * indicated *p* ≤ 0.05, ** *p* ≤ 0.01, *** *p* ≤ 0.001 and *ns* means not significant relative to the optimal group in each test.

As shown in Figure 3a, under a NaOH concentration of 3 g/L, the K/S value of the fabric dyed with C.I. Reactive Black 5 gradually increased with increasing GTA concentration, reaching a peak of 14.17 at a GTA concentration of 40 g/L. When the GTA concentration exceeded 40 g/L, the K/S value began to decrease. This decline is attributed to excessive GTA leading to saturated grafting on the cotton fabric surface. The excess GTA causes dye aggregation, preventing dye molecules from diffusing into the interior of the cotton fibers. As shown in Figure 3b, with a fixed GTA concentration of 40 g/L, the K/S value first increased and then decreased as the NaOH concentration increased, reaching a maximum of 18.96 at 1 g/L NaOH. This trend is due to residual NaOH in the modification solution increasing with higher NaOH concentrations, which promotes dye hydrolysis during the subsequent same-bath dyeing process. Figure 3c shows that, under the conditions of 40 g/L GTA and 1 g/L NaOH, the K/S value reached its maximum at a modification temperature of 90 °C. On one hand, higher temperatures may break the newly formed ether bonds, causing detachment of the modifier. On the other hand, excessive hydrolysis of epoxy groups leads to reduced reactivity of GTA. As shown in Figure 3d, under the conditions of 40 g/L GTA, 1 g/L NaOH, and a modification temperature of 90 °C, the K/S value of dyed fabric gradually increased and then stabilized with the extension of modification time. The K/S value of dyed fabrics after 50 min was 22.08. Therefore, the modification time of 50 min was selected as the optimal parameter for C.I. Reactive Black 5.

The class of chromophore/reactive groups in the structure of reactive dyes has a significant impact on their dyeing performance [47]. To investigate whether the SSFP yields comparable effects on other structural reactive dyes, anthraquinone-based C.I. Reactive Blue 19 and azo-based C.I. Reactive Red 195 were selected to validate the process outcomes. The results are shown in Figure 3e–l. Both reactive dyes showed good salt-free dyeing performance under the following conditions: GTA concentration of 40 g/L, NaOH concentration of 1 g/L, and modification temperature of 90 °C. Both Reactive Red 195 and Reactive Black 5 feature single azo structures, whereas Reactive Blue 19 possesses an anthraquinone structure; therefore, their structural differences necessitate distinct modification times. Consequently, Reactive Blue 19 achieves optimal dyeing results after only 40 min of modification, while Reactive Red 195 and Reactive Black 5 require 50 min to reach optimal dyeing performance. Table S1 lists the key parameters including the cationization agent and its concentration, presence/absence of intermediate washing and drying, and dyeing conditions. Based on the data summarized in Table S1 and Figure S2, it can be found that a higher GTA concentration and separate washing and drying steps are typically required [20,22,48,49]. This work significantly simplifies the process, reducing water and energy consumption and shortening the overall production time.

### 3.3. Optimization of Dyeing Parameters: Effects of Bath Ratio, Time, and Temperature

**Figure 4:**
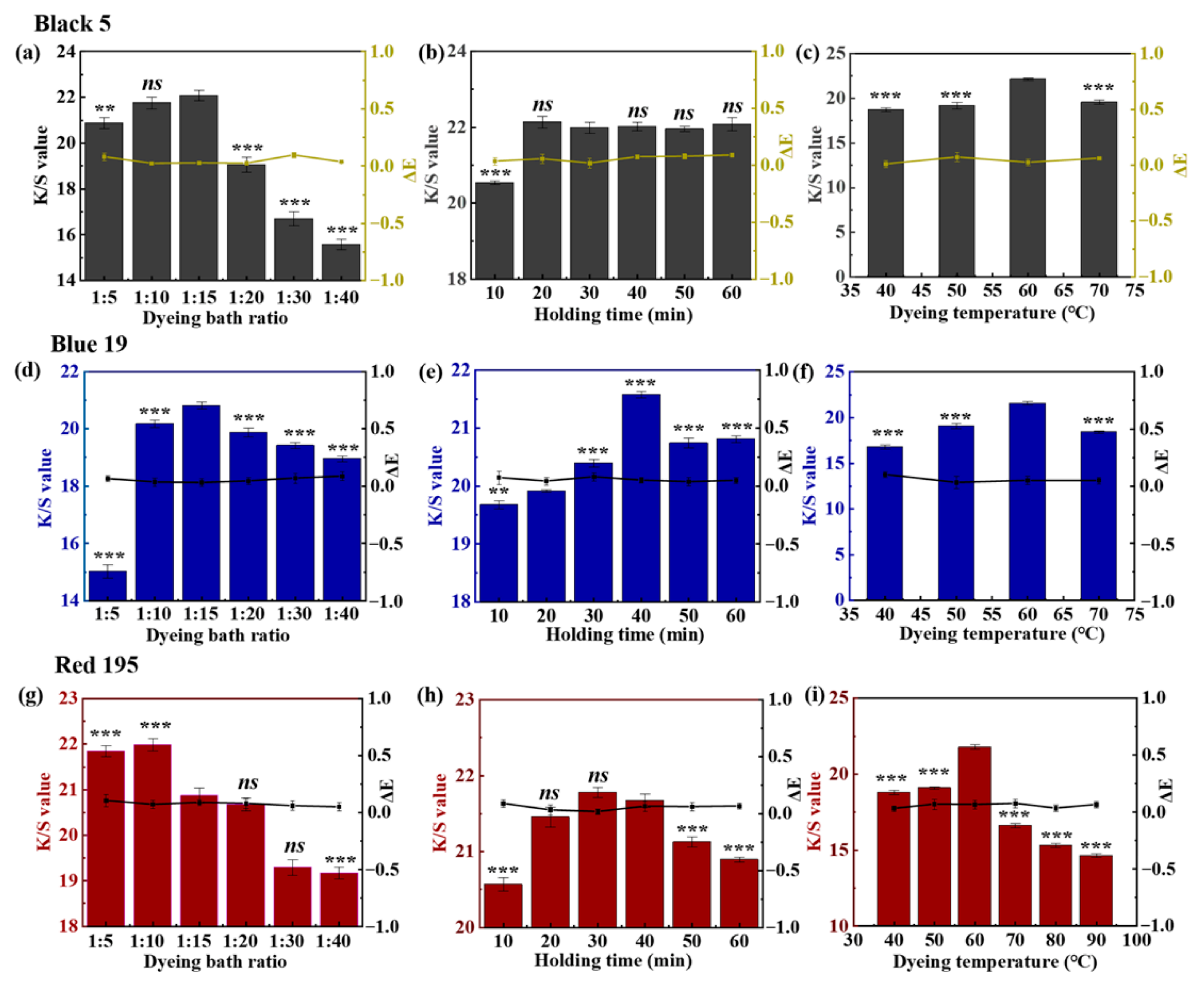
Effects of (**a**,**d**,**g**) different dyeing bath ratios, (**b**,**e**,**h**) holding times, and (**c**,**f**,**i**) dyeing temperatures on the dyeing performance of C.I. Reactive Black 5, Reactive Blue 19 and Reactive Red 195. ** indicated *p* ≤ 0.01, *** *p* ≤ 0.001 and *ns* means not significant relative to the optimal group in each test.

The effects of bath ratio, holding time, and temperature on the dyeing performance of three structurally distinct reactive dyes using the short-process cationization process were systematically investigated, as summarized in Figure 4. As shown in Figure 4, a bath ratio of 1:15 also yielded optimal dyeing performance for both Reactive Black 5 and Reactive Blue 19. Although no significant difference in the K/S value of Reactive Red 195 was observed between bath ratio of 1:5 and 1:10, it is worth noting that an excessively low dye solution volume may lead to insufficient fabric wetting and cause uneven dyeing. Therefore, a bath ratio of 1:15 was ultimately determined as optimal for all three dyes. Regarding holding time, C.I. Reactive Black 5, Reactive Blue 19 and Reactive Red 195 required 30, 20, and 40 min, respectively, to reach their optimal dyeing performance. The differences in the time required for the three reactive dyes to reach the dyeing equilibrium are essentially constrained by their molecular spatial steric hindrance and the diffusion rate in the amorphous region of the fibers. Among the dyes, C.I. Reactive Blue 19 has the simplest molecular structure, with extremely low spatial steric hindrance, thus possessing the optimal diffusion kinetics performance and being able to penetrate into the interior of the fibers most quickly. The diffusion rate of C.I. Reactive Black 5 is in the middle, and C.I. Reactive Red 195 showed the greatest migration resistance in the fiber micropores and the slowest diffusion due to its large molecular structure and long conjugated chain. Temperature optimization experiments revealed distinct behaviors among the three dyes. Both Reactive Black 5 and Reactive Blue 19, vinyl sulfone-type dyes, are more suited to low-temperature dyeing. However, excessively low temperatures significantly reduce their dye uptake rates. In contrast, although Reactive Red 195, an s-triazine reactive dye, exhibits good high-temperature adaptability, its K/S value decreases when the temperature exceeds 60 °C due to accelerated dye hydrolysis. Consequently, 60 °C was selected as the optimal temperature parameter for balancing dyeing efficiency and stability across all three dyes.

### 3.4. Investigation on the Bonding Mode Between Modified Cotton and Reactive Dyes

To investigate whether covalent bonding exists between modified cotton fabrics and dyes, the three dyes (C.I. Reactive Blue 19, Reactive Black 5, and Reactive Red 195) were subjected to hydrolysis. Subsequently, the modified cotton fibers were dyed using the hydrolyzed dyes in the SSFP. The presence of covalent bonds between the modified cotton fibers and the reactive dyes was determined by comparing the K/S values of the unhydrolyzed dyes and hydrolyzed dyes. To determine whether the reactive dyes underwent complete hydrolysis, the hydrolysis profiles of the three dyes and their HPLC analysis are presented in the Supporting Information (Figures S3 and S4).

**Figure 5:**
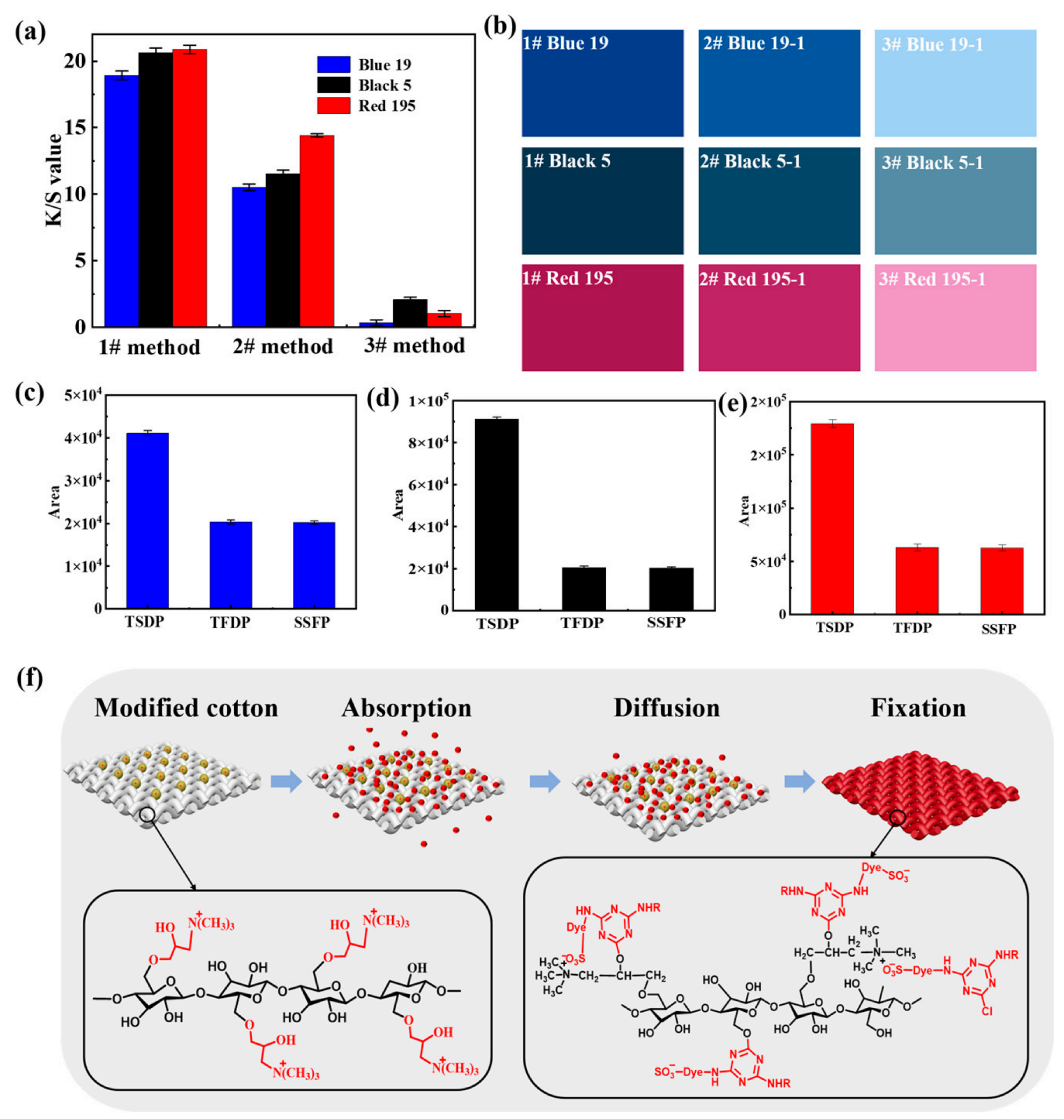
(**a**) K/S values, (**b**) images of dyes under the conditions (1#: cotton dyed with Blue 19/Black 5/Red 195 in SSFP, 2#: cotton dyed with Blue 19-1/Black 5-1/Red 195-1 in SSFP, 3#: cotton dyed with Blue 19-1/Black 5-1/Red 195-1 in TSDP); (**c**–**e**) residual dye content in the residual solutions of Reactive Blue 19, Reactive Black 5, and Reactive Red 195 after dyeing; (**f**) bonding mode between reactive dyes and modified cotton.

It can be clearly found in Figure 5a,b that, when reactive dyes were used for the SSFP, the K/S values of the fabrics dyed with C.I. Reactive Blue 19, Reactive Black 5, and Reactive Red 195 were 18.92, 20.63, and 20.88, respectively. They were significantly higher than those (10.53, 11.55, and 14.42) when hydrolyzed dyes were used for the SSFP. When hydrolyzed dyes were used for the TSDP (with salt and alkali), the K/S values of dyed fabrics were only 0.34, 2.07, and 1.03, respectively, indicating that the dyes were almost completely hydrolyzed. Here the dye hydrolysis process was analyzed using HPLC as shown in Figures S3 and S4. As shown in Figure 5c–e, HPLC analysis of the residual solutions after three dyeing methods showed that the residual dye content of the TSDP for C.I. Reactive Blue 19, Reactive Black 5, and Reactive Red 195 was higher than that of cationic dyeing, indicating that the cationization of cotton fibers improved the dye utilization rate. Moreover, the residual dye contents of the SSFP and the TFDP were very close. Figure 5f demonstrates that reactive dyes can firmly bond with cationic modified cotton fabrics in two ways: ionic bonds and covalent bonds. As a result, the SSFP not only saves time and energy but also achieves the same effect as conventional cationic dyeing. It provides a new approach for salt-free dyeing of cotton fabrics in the future.

### 3.5. Comparison of Overall Performance for the Three Different Dyeing Methods

**Figure 6:**
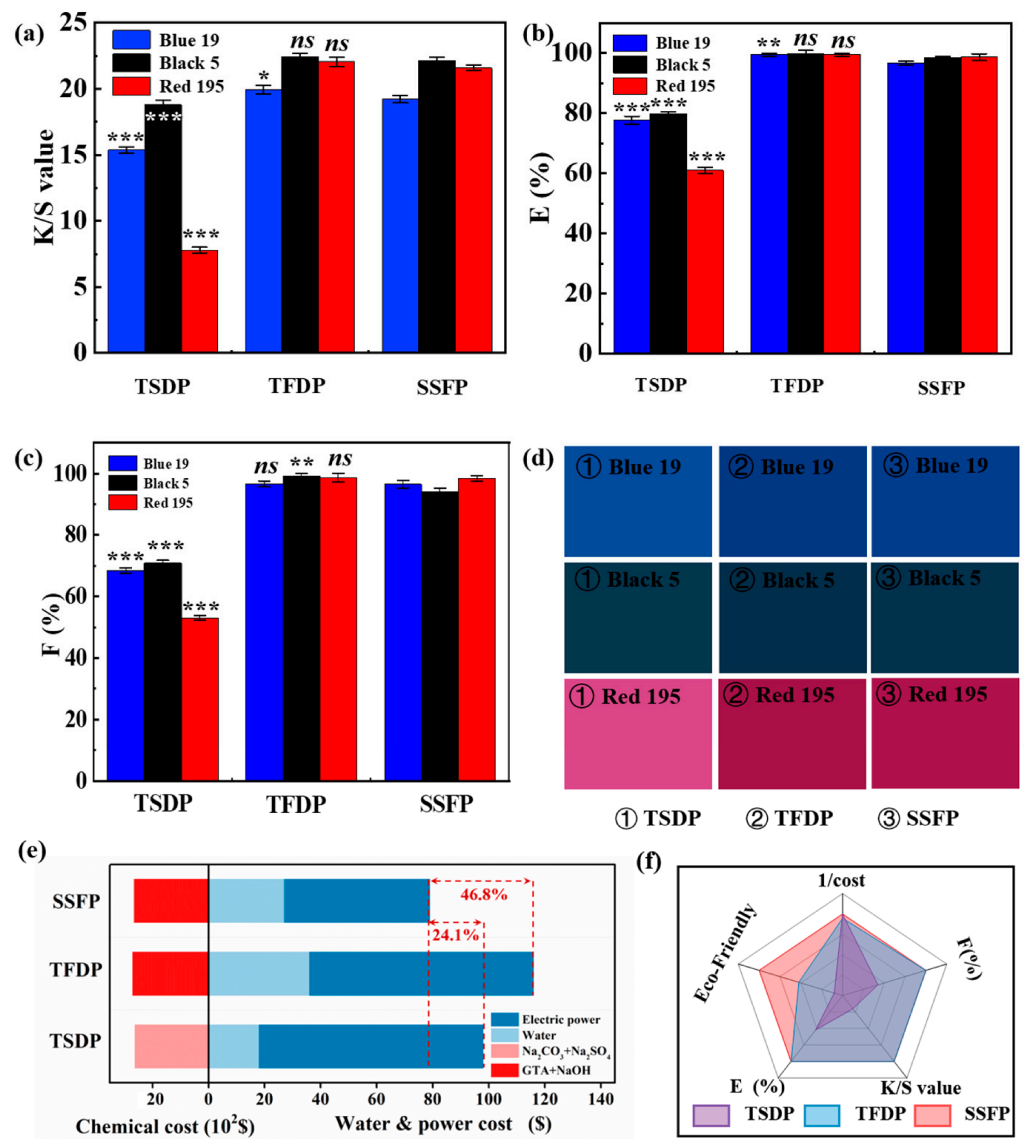
(**a**) K/S values, (**b**) dye uptake rates, (**c**) fixation rates of the three dyes under different process conditions; (**d**) images of dyed fabrics. (**e**) Cost breakdown of chemicals, water, and electricity for the three dyeing processes. (**f**) A comparative analysis of the SSFP against the TSDP and the TFDP was conducted in terms of cost, environmental friendliness, dyeing rate (E%), K/S value, and color fixation rate (F%). The results highlight the overall advantages of the SSFP. * indicated *p* ≤ 0.05, ** *p* ≤ 0.01, *** *p* ≤ 0.001 and *ns* means not significant relative to the optimal group in each test.

As shown in Figure 6a–c, under the TSDP, the K/S values of the fabrics dyed with C.I. Reactive Blue 19, Reactive Black 5, and Reactive Red 195 were 15.37, 18.80, and 7.79, respectively. And their fixation rates were 68.49%, 70.88% and 53.07%, respectively. After traditional cationic modification of cotton fabrics, the K/S values of the three dyes were 19.93, 22.42, and 22.04 respectively. Meanwhile, the fixation rates of the dyes reached 99.12%, 99.41% and 98.61%, respectively, indicating that the dyes were fully utilized. Under the SSFP, the K/S values of the three dyes were 19.22, 22.14, and 21.58, respectively, and the corresponding dye uptake rates and fixation rates were close to those of the TFDP. However, the K/S value, dye uptake rate, and fixation rate of cotton fabrics obtained by the SSFP were comparable to those obtained by the TFDP (modification–drying–dyeing). Images of fabrics dyed using the three different methods are shown in Figure 6d. The color appearance obtained from the SSFP is similar to that obtained from the TFDP. The color fastness of cotton fabrics dyed by the different methods is presented in Table S3. Compared with the conventionally dyed fabrics, the cationically modified samples exhibited a slight reduction in color fastness. The extent of the decrease varied among the three reactive dyes: C.I. Reactive Blue 19 showed the smallest decline (from Grade 4 to Grade 3~4), followed by C.I. Reactive Black 5 (from Grade 3~4 to Grade 3), and C.I. Reactive Red 195 showed the largest drop (from Grade 4 to Grade 3). Notably, the color fastness of the SSFP-dyed fabrics was essentially the same as that of the TFDP-dyed fabrics, indicating that the elimination of intermediate steps did not compromise this property.

Although the color fastness of cationically dyed fabrics is slightly lower than that of conventionally dyed fabrics, the SSFP offers significant advantages in terms of time savings, energy efficiency, water conservation, and environmental friendliness. As shown in Figure 6e and Tables S3 and S4, the chemical cost difference between the TSDP, the TFDP and the SSFP may appear modest. Although the per-ton cost saving may appear incremental, the cumulative economic, operational, and environmental benefits at industrial scale are by no means negligible for the SSFP. First, it is estimated that there are nearly 24.1% and 46.8% reductions in water and electricity expenses compared with the TSDP and the TFDP, respectively, which translates into considerable annual savings for a typical dyeing mill (Figure 6e). Second, beyond the direct monetary savings, the SSFP offers additional operational advantages that are not fully captured by cost figures alone. The elimination of the intermediate washing and drying steps may not only shorten the total processing time but also reduce equipment occupancy and labor requirements, thereby improving overall production efficiency and throughput. These indirect benefits are particularly valuable in industrial settings where production scheduling and capacity utilization are critical to profitability. Third, from an environmental compliance perspective, the reduction in wastewater generation and salt discharge alleviates the burden on downstream wastewater treatment facilities. As increasingly stringent environmental regulations are being enforced worldwide, the SSFP offers a more sustainable solution that helps mills avoid potential fines or costly upgrades to their treatment infrastructure. This regulatory advantage, while not immediately reflected in the cost comparison, represents a significant long-term economic and strategic benefit. Collectively, the cost-effectiveness of the SSFP, along with its superior environmental friendliness, dyeing rate (E%), K/S value, and color fixation rate (F%), positions it as a sustainable and highly competitive alternative for cotton dyeing (Figure 6f).

## 4. Conclusions

In this study, we developed a short-process cationic dyeing method for cotton fabrics to address the issues of lengthy procedures, high water consumption, and environmental pollution associated with conventional cationic dyeing processes. Using GTA as the cationic modifier, we successfully achieved salt-free dyeing of cotton fabrics in a simplified and water-saving manner. The optimal dyeing conditions for three structurally distinct reactive dyes were determined as follows: GTA concentration of 40 g/L, NaOH concentration of 1 g/L, modification temperature of 90 °C, dyeing bath ratio of 1:15, and dyeing temperature of 60 °C. The optimal modification time varied depending on dye structure: 40 min for C.I. Reactive Blue 19 (anthraquinone type), and 50 min for C.I. Reactive Black 5 and Reactive Red 195 (both azo type). Regarding holding time, C.I. Reactive Red 195, Reactive Black 5, and Reactive Blue 19 required 30, 20, and 40 min, respectively, to achieve optimal performance. After modification, the thermal stability and crystallinity of the cotton fabric decreased slightly (weight loss temperature from 300 °C to 270 °C; crystallinity from 60.51% to 58.18%), while its hydrophilicity remained unchanged. Mechanistic analysis confirmed that the reactive dyes bond with the modified cotton fabrics through both ionic and covalent bonds. Importantly, the color fastness achieved by the SSFP was identical to that of conventional cationic dyeing. This study provides a short-process, salt-free, and water-saving cationic dyeing strategy for cotton fabrics that achieves dyeing performance comparable to conventional methods while significantly reducing processing time and environmental impact. The proposed SSFP also shows good potential for industrial application, with simplified processing steps compatible with continuous dyeing lines, though scale-up challenges such as process stability and reproducibility remain to be further optimized in future pilot-scale trials. In summary, this work offers a promising new approach for the sustainable cationic dyeing of cotton fabrics in the future.

## Figures and Tables

**Table 1:** Gradient elution system for C.I. Reactive Red 195.

Time/min	Solvent/A%	Solvent/B%
0	80	20
14	75	25
15	80	20

**Table 2:** Gradient elution system for C.I. Reactive Black 5.

Time/min	Solvent/A%	Solvent/B%
0	80	20
18	63	37
15	80	20

**Table 3:** Gradient elution system for C.I. Reactive Blue 19.

Time/min	Solvent/A%	Solvent/B%
0	80	20
20	60	40
30	80	20

## Data Availability

The raw data supporting the conclusions of this article will be made available by the authors on request.

## Supplementary Materials

The following supporting information can be downloaded at https://www.mdpi.com/article/10.3390/polym18151867/s1: Figure S1: (a) Reactive Black 5; (b) Reactive Blue 19; (c) Reactive Red 195; (d) GTA. Figure S2: Comparison of two salt-free processes. Figure S3: (a) Hydrolysis Process of Reactive Red 195; (b) Hydrolysis Process of Reactive Blue 19; (c) Hydrolysis Process of Reactive Black 5. Figure S4: (a) Hydrolysis chromatogram of Reactive Blue 19; (b)Hydrolysis chromatogram of Reactive Black 5; (c)Hydrolysis chromatogram of Reactive Red 195. Table S1: The nitrogen content (N%) before and after GTA modification from the EDS elemental mapping results. Table S2: Color fastness of reactive dyes with different structures. Table S3: Comparison of representative cationic dyeing approaches. Table S4: Quantitative estimates of chemical and water consumption and their associated costs for TSDP, TFDP, and SSFP. Table S5: Electric power used for per ton of dyed fabric.
