# Supplementary material for: Cotton Cationization and Dyeing in One Bath: A Short-Process, Salt-Free Approach for Reactive Dyes
Source: Polymers (Basel). 2026 Jul 30;18(15):1867. doi: 10.3390/polym18151867 (PMC13468682; doi:10.3390/polym18151867)
Supplement: Supplementary file 1 [file polymers-18-01867-s001.zip › polymers-4429532-supplementary.pdf]

Cotton Cationization and Dyeing in One Bath: A Short-Process, Salt-Free Approach for Reactive Dyes

Zijian Dai [1], Jiao Li [1], Zixian Zhang [2], Huiqiang Wang [2], Jianyong Yu [1], Hongjuan Zhang [2,*], and Yang Si [1,3*]

1. *Innovation Center for Textile Science and Technology, Donghua University, Shanghai 200051, China*

2. *School of Textile and Fashion; Shanghai University of Engineering Science, Shanghai, 201620, P.R, China*

3. *State Key Laboratory for Modification of Chemical Fibers and Polymer Materials, Donghua University, Shanghai 201620, China*

* Corresponding author：hjz@sues.edu.cn; yangsi@dhu.edu.cn

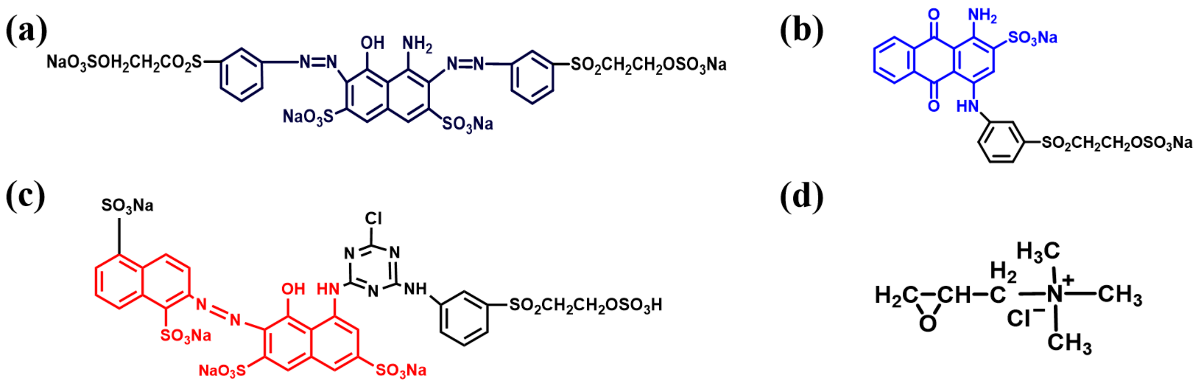

Figure S1. (a) Reactive Black 5; (b) Reactive Blue 19; (c) Reactive Red 195; (d) GTA

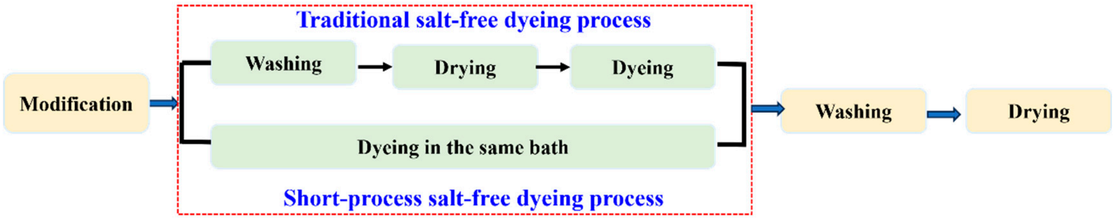

Figure S2. Comparison of two salt-free processes.

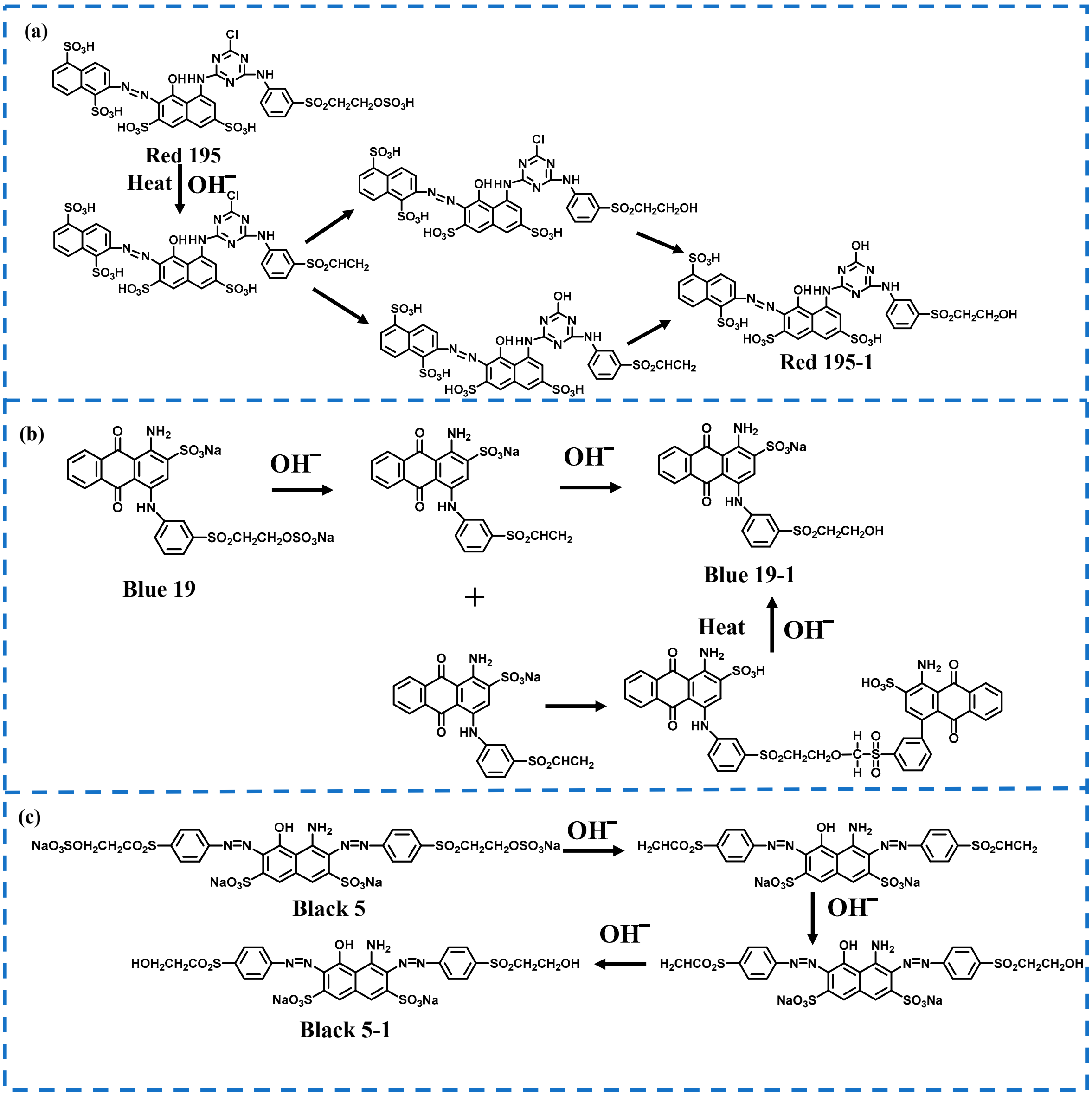

Figure S3. (a) Hydrolysis Process of Reactive Red 195; (b) Hydrolysis Process of Reactive Blue 19; (c) Hydrolysis Process of Reactive Black 5.

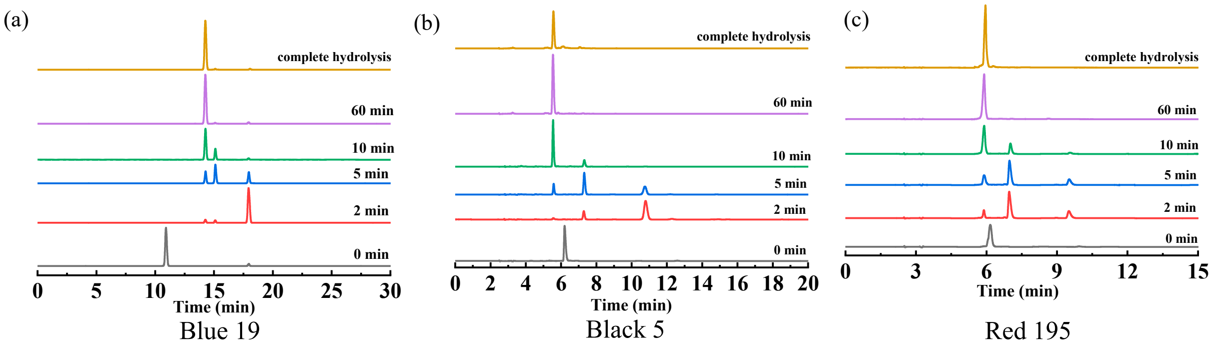

Figure S4 (a) Hydrolysis chromatogram of Reactive Blue 19; (b)Hydrolysis chromatogram of Reactive Black 5; (c)Hydrolysis chromatogram of Reactive Red 195.

Table S1. The nitrogen content (N%) before and after GTA modification from the EDS elemental mapping results.

Element	Raw cotton (%)	GTA-modified cotton (%)
N	0	1.12

Table S2. Color fastness of reactive dyes with different structures

Dye	Process	Washing Fastness						Rubbing Fastness	
		Wool	Acrylic	Polyester	Nylon	Cotton	Acetate	Dry	Wet
Blue 19	①	4	5	5	4-5	4-5	5	5	4
	②	4	5	5	4	4	5	5	3-4
	③	4	5	5	4	4	5	5	3-4
Black 5	①	4-5	5	5	5	5	5	5	3-4
	②	4-5	5	5	5	5	5	5	3
	③	4-5	5	5	5	5	5	5	3
Red 195	①	5	5	5	5	4-5	5	5	4
	②	5	5	5	5	4-5	5	5	3
	③	5	5	5	5	4-5	5	5	3

①Traditional Salt-Containing Dyeing Process (TSDP); ②Traditional Salt-Free Dyeing Process (TFDP); ③Short-Process Salt-Free Dyeing Process (SSFP)

Table S3. Comparison of representative cationic dyeing approaches.

Cationization agent and concentration	Intermediate washing & drying after cationization (Yes/No)	Dyeing condition	Reference
Glycidyltrimethylammonium chloride (GTA), 100 g/L	Yes	30-50 °C, 60 min	*Int. J. Biol. Macromol.*, 2024, 282, 136992
GTA, 55 g/L	Yes	30 °C, 30 min; 60 °C, 60 min	*Polymers*, 2022, 14, 5546
3-chloro-2-hydroxypropyltrimethylammoinium chloride, 50, 75, 100 g/L	Yes	30-40-50 °C, 60 min	*Dyes & Pigments*, 2026, 246, 113381
3-chloro-2-hydroxypropyl trimethyl ammonium chloride, polydiallyldimethyl ammonium chloride, polyethyleneimine, 3 g/L	Yes	70 °C, 30 min; Na_2CO_3 (1g/L), 20 min	*Green Chem.*, 2022, 24, 9180
GTA, 40 g/L	No	60 °C, 20-40 min	This work

Table S4. Quantitative estimates of chemical and water consumption and their associated costs for TSDP, TFDP, and SSFP.

Process	Chemical	Average price ($/t)	TSDP		TFDP		SSFP	
			Consumption (t)	Cost ($)[b]	Consumption (t)	Cost ($)	Consumption (t)	Cost ($)
Modification	GTA	4386.75	/		0.6	2632.05	0.6	2632.05
	NaOH	1813.05	/		0.045	81.587	0.015	27.195
	Water	0.6[a]	/		15	9	15	9
Washing	Soap	1909.6	/		0.03	57.29	/	
	Water	0.6[a]	/		15	9	/	
Dyeing	Dyes	3172	0.03	95.16	0.03	95.16	0.03	95.16
	Na_2CO_3	2293.21	0.3	687.963	/		/	
	Na_2SO_4	2162.29	0.9	1946.061	/		/	
	Water	0.6[a]	15	9	15	9	15	9
Washing	Soap	1909.6	0.03	57.29	0.03	57.29	0.03	57.29
	Water	0.6[a]	15	9	15	9	15	9
Total	Water		30	18	60	36	45	27
	Chemical Cost ($)		/	2786.474	/	2923.377	/	2811.695
Sustainability	Wastewater (t)		30	/	/		/	
	Water saving (t)		/	/	60	/	45	/

Table S5. Electric power used for per ton of dyed fabric

	Unit price (USD/kWh)	TSDP (kWh)	TFDP (kWh)	SSFP (kWh)
Electric power	0.1	800	800	520
